# Peripheral Vasospasm Following Jellyfish Envenomation: A Case Report

**DOI:** 10.7759/cureus.107147

**Published:** 2026-04-16

**Authors:** Ibraheem Saleh

**Affiliations:** 1 Emergency Medicine, The View Hospital, Doha, QAT

**Keywords:** calcium-channel blockers, emergency medicine, jellyfish envenomation, marine toxins, raynaud phenomenon, venom-induced vasospasm

## Abstract

Jellyfish stings are a common marine injury worldwide, most often resulting in localized cutaneous manifestations. Delayed vascular complications are rarely reported. We describe the case of a 31‑year‑old previously healthy woman who developed delayed peripheral vasospasm following a jellyfish sting to the dorsum of her left hand. Several days after the initial injury, she presented with painful bluish discoloration and cold sensitivity affecting the ring and little fingers. Clinical evaluation revealed preserved distal perfusion with findings consistent with peripheral vasospasm resembling secondary Raynaud phenomenon. Management included topical glyceryl trinitrate and the initiation of a calcium‑channel blocker, resulting in significant symptomatic improvement. This case highlights an uncommon vascular sequela of jellyfish stings and emphasizes the importance of recognizing delayed vascular complications in emergency settings.

## Introduction

Jellyfish stings represent one of the most common marine injuries worldwide, accounting for millions of stings annually, particularly in coastal and tropical regions. Most cases are self‑limited and characterized by localized pain, erythema, and urticarial skin reactions. However, systemic and delayed complications, though uncommon, have been described and may pose significant diagnostic and therapeutic challenges.

Peripheral vasospasm following jellyfish stings is rarely reported but is thought to result from toxin‑mediated endothelial dysfunction and vasomotor instability. Raynaud‑like phenomena triggered by jellyfish stings represent an uncommon yet clinically significant presentation that requires prompt recognition to prevent ischemic complications and tissue injury [1,2]. Awareness of such delayed vascular complications is particularly important for clinicians practicing in coastal regions or emergency settings with regular marine exposure.

We report a rare case of delayed peripheral vasospasm following a jellyfish sting, highlighting the clinical course, vascular assessment, management strategy, and relevance to emergency practice.

## Case presentation

A 31‑year‑old previously healthy woman, with no significant past medical history, no regular medications, and no known drug allergies, sustained a jellyfish sting to the dorsum of her left hand while swimming in a saltwater marine environment. The exact species could not be identified, as no photographs were available and the patient was unable to describe the jellyfish in detail.

**Figure 1:**
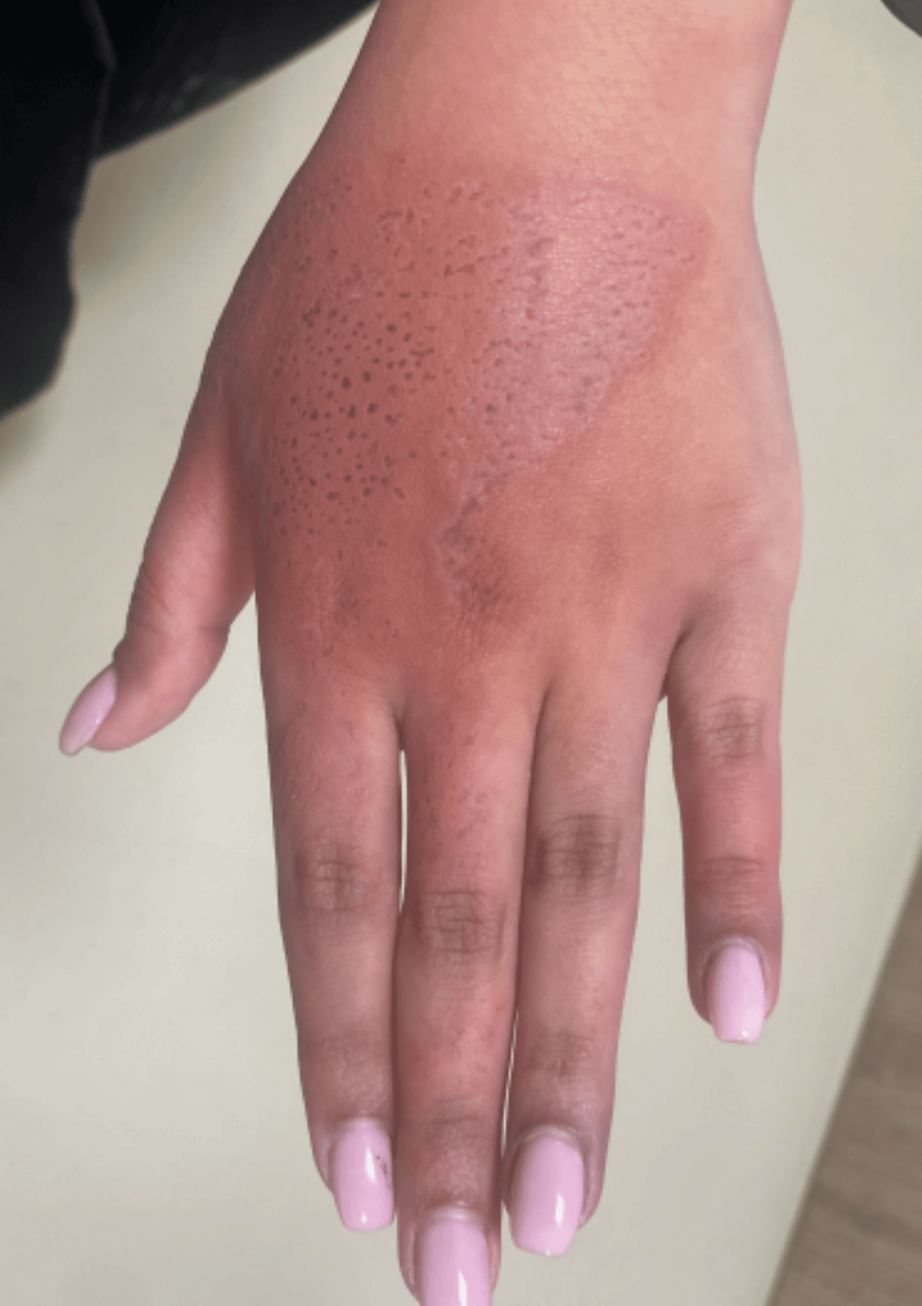
Dorsal aspect of the left hand showing persistent erythema at the site of the jellyfish sting

Within hours of the sting, she developed localized erythema and swelling at the sting site (Figure 1). She initially presented to a peripheral healthcare facility, where she was treated symptomatically with oral antihistamines and paracetamol and discharged without further investigations.

**Figure 2:**
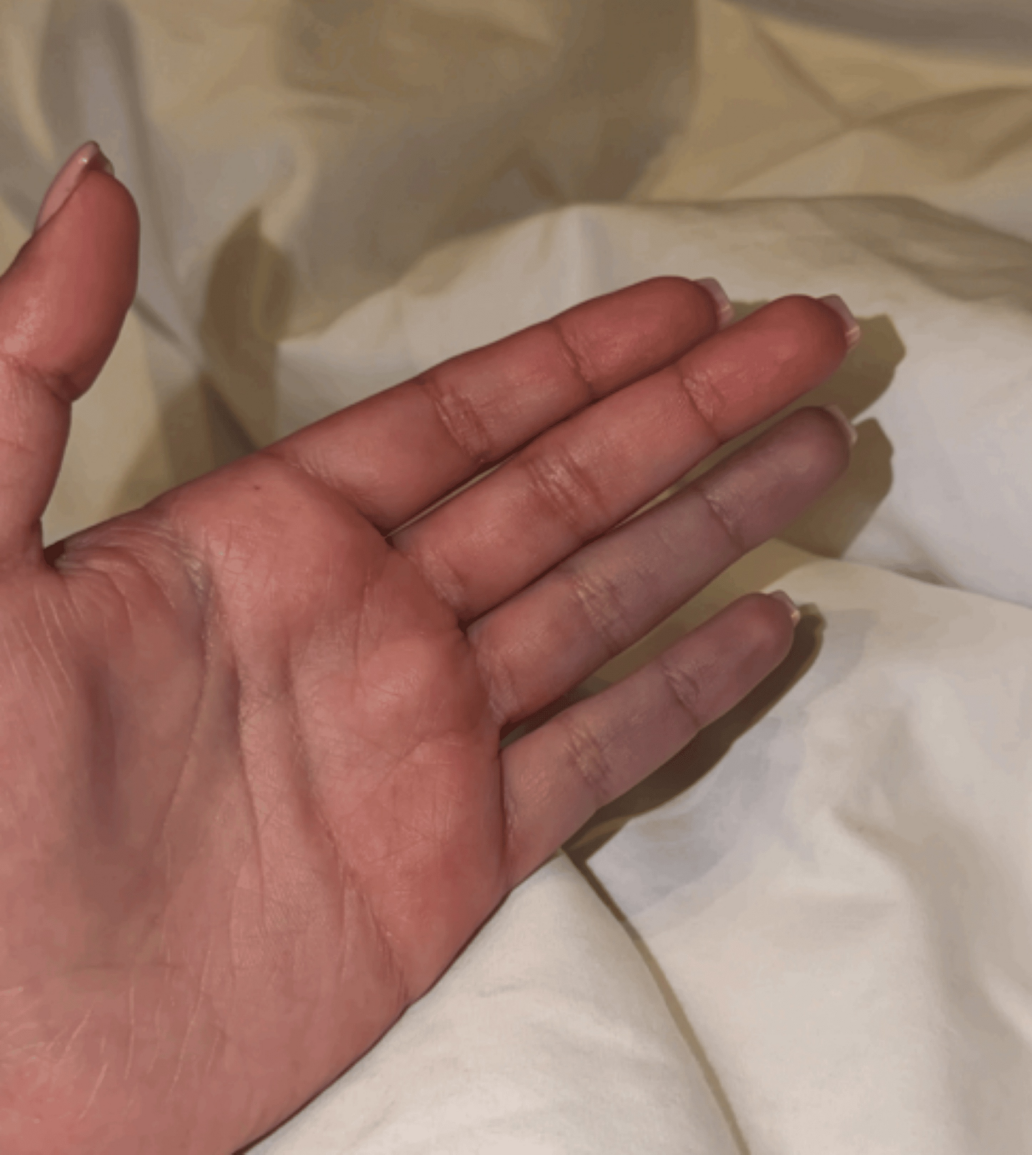
Bluish discoloration of the left ring and little fingers following jellyfish sting, consistent with peripheral vasospasm

Approximately five days after the sting, the patient developed progressive discoloration of the left ring and little fingers. She reported persistent tenderness, burning pain, and marked hypersensitivity to cold exposure. On examination, the affected digits appeared bluish and cold to touch (Figure 2), while erythema at the sting site on the dorsum of the hand remained present (Figure 1). These symptoms persisted for approximately three days prior to re‑presentation to a tertiary care emergency department.

At re‑presentation, distal pulses were palpable, capillary refill was preserved, and no sensory or motor deficits were identified. Findings were consistent with peripheral vasospasm rather than fixed arterial obstruction. No additional diagnostic testing was performed at this visit, as the diagnosis was made clinically based on characteristic features and physical examination.

Topical glyceryl trinitrate 0.4% ointment was initiated, applied to the affected digits twice daily, and the patient was referred for rheumatology evaluation. Eight days after the initial sting, she was assessed by a consultant rheumatologist and diagnosed with secondary Raynaud syndrome. Amlodipine 5 mg orally once daily was initiated as a temporary measure, with plans for reassessment and discontinuation upon symptom resolution.

Basic laboratory evaluation for autoimmune etiologies was considered but not completed due to significant clinical improvement. Doppler ultrasonography was suggested but not pursued, as palpable pulses, preserved capillary refill, and rapid response to vasodilator therapy made significant arterial occlusion unlikely.

During the follow‑up telephone review approximately 10 days later, the patient reported marked improvement in pain, discoloration, and cold sensitivity, with no progression or recurrence of symptoms.

## Discussion

Although most jellyfish stings result in benign local reactions, severe vascular complications have been reported, ranging from Raynaud‑like vasospasm to digital ischemia and, in rare cases, gangrene. Binnetoglu et al. described digital necrosis following a jellyfish sting in a pediatric patient, emphasizing the potential severity of toxin‑mediated vasospastic responses even in individuals without underlying connective tissue disease [1].

Several reports have described the use of intravenous prostacyclin analogs such as iloprost for severe or refractory jellyfish‑associated digital ischemia [2,3]. While these therapies may be effective in advanced cases with threatened tissue viability, they were not required in the present case.

In contrast, our patient demonstrated significant improvement with topical glyceryl trinitrate and oral calcium‑channel blockade, highlighting the effectiveness of conservative, readily available vasodilator therapies when vasospasm is recognized early. This aligns with the proposed pathophysiology of jellyfish stings, in which venom components, including vasoactive peptides, neurotoxins, and catecholamine‑like substances, may induce endothelial dysfunction and intense vasoconstriction, resulting in delayed vasospasm rather than fixed vascular occlusion.

The exact jellyfish species could not be identified in this case. However, species known to cause severe vascular effects, including box jellyfish (*Chironex *spp.) and the Portuguese man‑of‑war (*Physalia physalis*), are encountered in various marine regions worldwide. This underscores the importance of clinical vigilance regardless of confirmed species identification.

## Conclusions

Peripheral vasospasm is a rare but important delayed complication of jellyfish stings. Emergency physicians should maintain a high index of suspicion in patients presenting with Raynaud‑like symptoms following marine exposure. Careful vascular examination and timely initiation of vasodilator therapy can lead to rapid symptom resolution and prevent progression to ischemic injury.
